# The metabolic core of the prokaryotic community from deep-sea sediments of the southern Gulf of Mexico shows different functional signatures between the continental slope and abyssal plain

**DOI:** 10.7717/peerj.12474

**Published:** 2021-12-14

**Authors:** Mónica Torres-Beltrán, Lluvia Vargas-Gastélum, Dante Magdaleno-Moncayo, Meritxell Riquelme, Juan Carlos Herguera-García, Alejandra Prieto-Davó, Asunción Lago-Lestón

**Affiliations:** 1 Departamento de Innovación Biomédica, Centro de Investigación Científica y de Educación Superior de Ensenada, Ensenada, Baja California, Mexico; 2 Departamento de Microbiología, Centro de Investigación Científica y de Educación Superior de Ensenada, Ensenada, Baja California, Mexico; 3 Facultad de Ingeniería, Arquitectura y Diseño, Universidad Autónoma de Baja California, Ensenada, Baja California, Mexico; 4 Departamento de Ecología Marina, Centro de Investigación Científica y de Educación Superior de Ensenada, Ensenada, Baja California, Mexico; 5 Facultad de Química, Universidad Nacional Autónoma de México, Sisal, Yucatán, Mexico

**Keywords:** Microbial community metabolism, Deep-sea sediments, Hydrocarbons, Amino acids, Gulf of Mexico, Metagenomics

## Abstract

Marine sediments harbor an outstanding level of microbial diversity supporting diverse metabolic activities. Sediments in the Gulf of Mexico (GoM) are subjected to anthropic stressors including oil pollution with potential effects on microbial community structure and function that impact biogeochemical cycling. We used metagenomic analyses to provide significant insight into the potential metabolic capacity of the microbial community in Southern GoM deep sediments. We identified genes for hydrocarbon, nitrogen and sulfur metabolism mostly affiliated with Alpha and Betaproteobacteria, Acidobacteria, Chloroflexi and Firmicutes, in relation to the use of alternative carbon and energy sources to thrive under limiting growth conditions, and metabolic strategies to cope with environmental stressors. In addition, results show amino acids metabolism could be associated with sulfur metabolism carried out by Acidobacteria, Chloroflexi and Firmicutes, and may play a crucial role as a central carbon source to favor bacterial growth. We identified the tricarboxylic acid cycle (TCA) and aspartate, glutamate, glyoxylate and leucine degradation pathways, as part of the core carbon metabolism across samples. Further, microbial communities from the continental slope and abyssal plain show differential metabolic capacities to cope with environmental stressors such as oxidative stress and carbon limiting growth conditions, respectively. This research combined taxonomic and functional information of the microbial community from Southern GoM sediments to provide fundamental knowledge that links the prokaryotic structure to its potential function and which can be used as a baseline for future studies to model microbial community responses to environmental perturbations, as well as to develop more accurate mitigation and conservation strategies.

## Introduction

Biogeochemical processes in marine sediments can be related to the degradation of organic matter via multiple metabolic reactions involving a myriad of organisms, oxidants and intermediate compounds that result in most of the carbon eventually respired as needed for mass-balance closure (Arndt et al., 2013; Middelburg, 2018). Organic matter in the ocean is generally a mixture of different compounds including carbohydrates, amino acids, lipids, and a large portion of uncharacterized chemically complex compounds resistant to biological degradation (Burdige, 2007; Hedges & Oades, 1997; Schnitzer, 1999). Overall, the downward flux of organic matter and its delivery to the seafloor depends on sedimentation rates of sinking particles that mostly vary as a function of primary production in the ocean surface, and of the proximity to continental sources (Arndt et al., 2013; Middelburg, 2018; Orsi, 2018). Oxic deep-sea seafloor sediments underlie waters with low rates of organic matter sedimentation that tend to be far from the continental slope (between 4,000 and 6,000 m depth) and where primary productivity is limited by nutrient availability *i.e.*, abyssal plains (D’Hondt et al., 2015; Kallmeyer et al., 2012). In the abyssal plains, although oxygen penetrates very deeply, microbial communities are under extreme energy limitation due to the low amount of organic matter and electron donors from the overlying oligotrophic waters (reviewed in Orsi, 2018). Nonetheless, organic matter degradation processes result from the combined metabolic effort of billions of individual microorganisms. This results in microbial communities with unique metabolic strategies to cope for the limiting growth conditions, with a myriad of metabolic pathways to meet their growth requirements, and that are continuously involved in synergetic or competitive interactions that determine the outcome of their activities (Arndt et al., 2013; Middelburg, 2018). Thus, understanding microbial communities’ gene potential in these environments could be used to understand their metabolic capabilities and predict their response to environmental perturbations.

The Gulf of Mexico (GoM) is one of the most economically and ecologically productive and important bodies of water in the world (Fautin et al., 2010; Tunnell Jr, 2009; Yoskowitz et al., 2013). Historically, the GoM has been subjected to different environmental perturbations and chronic stressors, *i.e.*, urban and agricultural runoffs, commerce and transportation activities, and oil and gas industry activities (reviewed in Peterson et al., 2011; Ward & Tunnell, 2017), that have resulted in the continuous release of contaminants to the ecosystem. A wide range of contaminants have been found in the GoM sediments potentially altering the sensitive microbial communities’ metabolism (Urakawa et al., 2012; Yergeau et al., 2010; Yergeau et al., 2012) and, therefore, the biogeochemical processes related to organic matter degradation and its consequential carbon export. As the oil and gas industry in the GoM continues to expand in the search for new oil reserves in deep-marine waters, baseline environmental studies, including microbial community taxonomic and metabolic diversity, are of great interest for designing contingency plans and proposing mitigation strategies associated with oil-related environmental impacts *i.e.*, oil spills. For instance, after the Deepwater Horizon oil spill in 2010, most of the research efforts have focused on identifying impacts on microbial communities in the water column and sediments in the Northern region of the GoM (Bacosa et al., 2018; Bik et al., 2012; Godoy-Lozano et al., 2018; Kimes et al., 2013; Mason et al., 2014; Yergeau et al., 2015). To expand on this research, the oceanographic campaigns XIXIMI (traditional Nahuatl for “spill”) emerged as a Mexican initiative to characterize the oceanographic, hydrographic, biogeochemical and ecological baseline conditions throughout the entire Southern deep region of the GoM, in the deep-water region (those with depths >1,000 m) of Mexico’s Exclusive Economic Zone, to promptly detect any impacts in the water column and sediment ecosystems if an oil spill occurs over large spatial–temporal scales.

The geochemical characteristics for the GoM region have been reported (reviewed in Herguera García, Díaz Asencio & Cepeda Morales, 2021); however, it was through the XIXIMI campaigns that biogeochemical information of the Southern region started being generated. Surveys derived from the XIXIMI campaigns have characterized the geochemistry and microbiology of the deep-sea sediments of the Southern GoM. To date, microbiology surveys in Southern GoM have described the taxonomy of prokaryotic and eukaryotic communities (Godoy-Lozano et al., 2018; Vargas-Gastélum et al., 2019), setting the basis of the microbiota fingerprint in this region. Currently, research efforts are focused on generating environmental metagenomic information that enables a better understanding of the functional composition and potential of microbial communities in the Southern GoM deep-sea sediments (Raggi et al., 2020; Rodríguez-Salazar et al., 2021). Particularly, GoM deep-sea sediments have been reported to show exceptionally low average organic carbon values (<1%) compared to other deep slope and abyssal regions of the global ocean (Herguera García, Díaz Asencio & Cepeda Morales, 2021). Thus, the variety of metabolic strategies carried out by the microbial community in the sediments to cope with limiting growth conditions and its potential for hydrocarbon metabolism remains to be understood at these GoM regions. Generating fundamental knowledge on microbial communities is crucial to model or predict ecosystem response to possible environmental perturbations, particularly due to oil and gas industries’ expansion in this region. This research focuses on describing the microbial community’s potential for carbon transformation derived from metagenomic data generated from five sediment cores collected during the XIXIMI-03 campaign. Taxonomic and functional data was used to evaluate the prokaryotic community’s metabolic carbon core in the continental slope and abyssal plain in Southern GoM deep-sea sediments in a comprehensive manner, providing information on the most representative genes and metabolic pathways observed throughout the prokaryotic community, including those related with central metabolic pathways, and amino acids, hydrocarbons, nitrogen and sulfur metabolisms in hand with the taxonomic fingerprint of these processes.

## Materials & Methods

### Sample collection and geochemical analyses

Deep-sea sediment samples were collected during the XIXIMI-03 oceanographic campaign between February 9th and April 10th of 2013, on board the *R/V Justo Sierra*. Samples were collected using a multicorer at five different sampling stations: E2, E7, E44, E46 and E47, distributed along the Southern region of the GoM, and located on the continental slope and abyssal plain, with depths ranging from 1,739 to 3,741 m (Fig. S1). The sediment cores were subsampled for metagenomic analyses using a 1 mL-needleless sterilized syringe allowing to take the top first five cm of each core, and then transferred into 1.5 mL Eppendorf tubes containing sucrose buffer 25% wt/vol for sample preservation (Mitchell & Takacs-Vesbach, 2008). The tubes containing the sediments were stored at −20 °C until further processing.

For geochemical analyses, a different core liner from the multicorer was used. Cores were sliced at 1 cm intervals for the topmost 6 cm and then at two cm slices for the rest of the core, which were stored at −20 °C until processing. In the laboratory, samples were weighed while wet, freeze-dried and weighed again to calculate water content and porosity. For the carbonate content analysis dry samples were homogenized in an opal mortar, and a 15 mg aliquot of sediment was used to measure carbonate content by coulometry (UIC Coulometer CM5041) and calibrated with a pure carbonate reference and a laboratory reference CH94-94K. Content of carbonate is reported as a percentage with a precision of 0.1% (Abella-Gutiérrez & Herguera, 2016; Díaz-Asencio, Bartrina & Herguera, 2019). Terrigenous content (mostly silt and clay) was calculated assuming a constant concentration of 2% biogenic opal and a maximum content of organic carbon of 1% (Díaz-Asencio, Bartrina & Herguera, 2019).

### DNA extraction and sequencing

Sediments from the first five cm were used for DNA extraction and sequencing. Prior to DNA extraction, samples were centrifuged at 10,000× g for 1 min to precipitate the sample and discard the sucrose buffer. Genomic DNA was extracted from 0.25 gr of sediment using the PowerSoil DNA Isolation Kit (Mo Bio Laboratories Inc.) according to the manufacturer’s protocol. DNA was quantified via a NanoDrop lite (Thermo Fisher Scientific) spectrometer. Metagenome libraries were generated using the Nextera XT DNA Library Preparation Kit and sequencing was performed with the MiSeq Reagent Kit v3 (150-cycle) in an Illumina MiSeq platform.

### Bioinformatic and statistical analyses

Metagenomes were analyzed using the MetaGenome Rapid Annotation using Subsystems Technology (MG-RAST) on-line server (Keegan, Glass & Meyer, 2016), and are available in EMBL-EBI with the project ID PRJEB47540 and under the MG-RAST project ID mgp13888. In MG-RAST each unassembled metagenome was processed for quality control (QC) including sequences quality filtering (5 ambiguous base pairs maximum), length filtering (sequences with ± 2 standard deviations from the mean length were removed), and dereplication (removing of sequencing artifacts) (Kimes et al., 2013). Functional annotation was performed with a BLAT algorithm (Kent, 2002) search (maximum e-value of 0.001) using the non-redundant protein database M5NR (Wilke et al., 2012) and RefSeq (O’Leary et al., 2016). To further relate the functional and taxonomic composition with the metabolisms’ classification, a functional annotation table matching RefSeq taxonomic and functional assignments with SEED Subsystems classification for each sequence in metagenomes was constructed using the *dplyr* (Wickham et al., 2018) and *tidyr* (Wickham et al., 2019) packages in the R software (R Core Team, 2013). Gene data in the functional annotation table was normalized by the total number of reads in each sample and used in downstream analyses. Taxonomic and functional data visualization was conducted using the ggplot2 package (Wickham, 2009).

In order to infer the potential metabolic pathways carried out by the microbial community in sediments, we constructed environmental Pathway/Genome Databases (ePGDBs) using the MetaPathways “ePGDB” module, that uses the PathoLogic algorithm (Karp et al., 2010; Karp, Paley & Romero, 2002) to map functional annotations onto the MetaCyc (Caspi et al., 2016; Karp et al., 2000) collection of reactions and pathways. PathoLogic predicts metabolic pathways based on defined biochemical rules including pathway completion, diagnostic/key enzymes, biosynthesis and degradation constraints which results in ePGDB construction (Konwar et al., 2013). Environmental PGDBs were exported as .txt tables for further analysis. Hierarchical clustering analysis (HCA) was conducted on ePGDBs to identify community metabolic compositional profiles using the *pvclust* package in the R software (R Core Team, 2013; Suzuki, Terada & Shimodaira, 2015) with Manhattan Distance measure and statistical significance to the resulting clusters as bootstrap score distributions (1,000 iterations). In addition, we conducted a correlation analysis of environmental variables with metagenomic data clusters using the *envfit* function in the *vegan* package in the R software. Further, we identified the distinctive metabolic pathways in each of the metagenomes and those that were shared among all and were considered to constitute the core metabolism of the sediment’s microbial community. Metabolic pathways visualization was conducted using the *ggplot2* package (Wickham, 2009) and the *VennDiagram* package (Chen & Boutros, 2011) in the R software (R Core Team, 2013).

## Results

### Geochemical properties of Southern GoM deep-sea sediments

The geochemical composition of the sediments from the five sampling stations (E2, E7, E44, E46 and E47) was determined by the organic carbon, carbonate and terrigenous content (Table SI). Organic carbon content showed values ranging between 0.61–0.97%, with the highest value (0.97%) at E47 located at the continental slope of Yucatán, and lowest value (0.61%) at E44 located closer to the abyssal plain. Carbonate content showed values ranging between 21.9 and 38.03%, the highest value was observed for E47 (38.03%) and the lowest was observed for E2 (21.9%), located closer to the continental slope of Tamaulipas. Terrigenous content showed values ranging from 58.9–75.25%, the highest value observed at E2 and the lowest value was observed at E47.

### Microbial community taxonomic and functional composition in GoM deep sediments

To identify prokaryotic community gene composition, we analyzed genomic information comprised of 6,506,601 quality-filtered reads (Table SII) from five metagenomes corresponding to samples collected at stations E2, E7, E44, E46 and E47, during the XIXIMI-03 campaign.

**Figure 1:**
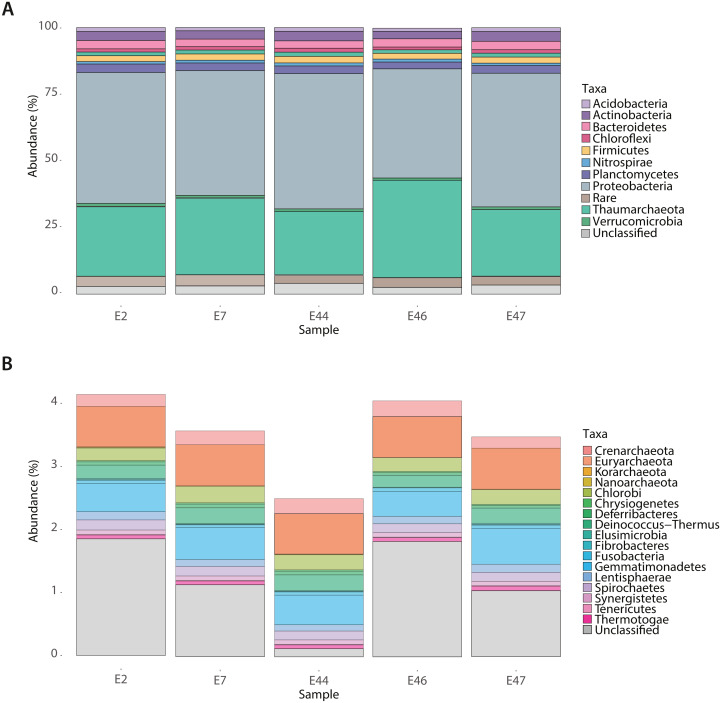
Taxonomic composition of the Southern GoM sediments. Bar plots depict the relative abundance (%) of identified microbial groups based on contig-LCA annotations of metagenomes (E2, E7, E44, E46 and E47) in MG-RAST. (A) Abundant (>1% relative abundance) microbial groups and (B) Rare (<1% relative abundance) microbial groups. Bar plot are divided and colored based on taxonomic affiliation as indicated in color key.

To identify the deep-sea sediments microbial community core among samples, the contigLCA taxonomic affiliation of genes, based on the M5NR database was used. The prokaryotic community core was constituted by taxa showing a relative abundance higher than 1% such as Acidobacteria, Actinobacteria, Bacteroidetes, Chloroflexi, Firmicutes, Nitrospirae, Planctomycetes, Proteobacteria, and Verrucomicrobia within the bacterial domain, and Thaumarchaeota within the archaeal domain (Fig. 1A). The Proteobacteria phylum was mostly constituted by Alphaproteobacteria affiliated with Caulobacterales, Rhodospirillales, Rhizobiales, Rickettsiales, Rhodobacterales and Sphingomonadales; Betaproteobacteria affiliated with Burkholderiales, Methylophilales, Neisseriales and Nitrosomonadales; Deltaproteobacteria affiliated with Desulfovibrionales and Desulfuromonadales; Epsilonproteobacteria affiliated with Campylobacterales; Gammaproteobacteria affiliated with Alteromonadales, Legionellales, Methylococcales, Oceanospirillales, Pseudomonadales, Pasteurellales, Thiotrichales, Vibrionales, and Xanthomonadales (Fig. S2). In comparison, the rare biosphere core was constituted by taxa showing a relative abundance lower than 1% including Chlorobi, Chrysiogenetes, Deferribacteres, Deinococcus-Thermus, Elusimicrobia, Fibrobacteres, Gemmatimonadetes, Lentisphaerae, Spirochaetes, Synergistetes and Thermotogae belonging to the bacterial domain, and Crenarchaeota, Euryarchaeota, Korarchaeota and Nanoarchaeota to the archaeal domain (Fig. 1B).

**Figure 2:**
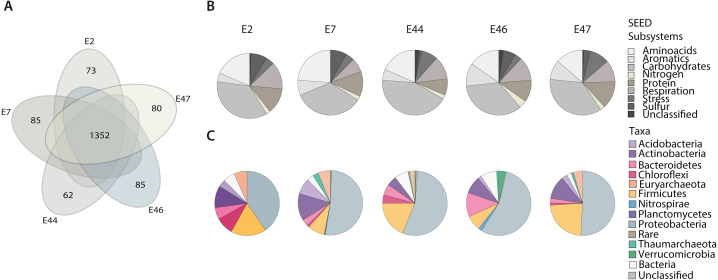
Gene classification and distribution in the Southern GoM sediments. (A) Five-way Venn-diagram depicts the number of shared (microbial community functional core) and unique genes observed in metagenomes (E2, E7, E44, E46 and E47). (B) Pie charts show the SEED-Subsystems classification of genes observed in metagenomes. (C) Pie charts show the taxonomic distribution of genes observed in metagenomes. Pie charts are divided based on abundance of genes related to a SEED-Subsystem or taxonomic group and colored as indicated in key.

To identify the deep-sea sediments prokaryotic community functional core using the SEED Subsystems classification (level 1) of genes across samples. We identified a total of 2,261 different functional genes (corresponding to 621,536 reads distributed among samples) within the SEED classes of metabolism, including Amino acids, Aromatics, Carbohydrates, Cell Wall and Capsule, Cofactors and Vitamins, DNA Metabolism, Fatty Acids, Lipids, and Isoprenoids, Nitrogen, Nucleosides and Nucleotides, Membrane Transport, Protein, Phages and Plasmids, RNA Metabolism, Respiration, Stress, Sulfur and Virulence (Fig. S3). Further, we identified the community’s functional core by cross comparing the gene content among metagenomes based on the RefSeq and SEED Subsystems annotations. Functional core was constituted by 1,352 genes (338,499 reads) (Fig. 2; Table SIII) representing 59% of the total genes identified mostly affiliated with Proteobacteria (44%) and Thaumarchaeota (10%). Unique genes were also found for each metagenome (Table SIV), representing between 4.6 and 5.8% of the total genes identified. These genes were distributed among the Amino acids, Aromatics, Carbohydrates, Nitrogen, Protein, Respiration, Stress and Sulfur SEED classes of metabolism (Fig. 2B) and showed differences in their abundance (expressed as % of the total number of genes in a sample) across samples. For instance, genes related to the Respiration class showed the highest abundance (13.5%) at E2, while genes related to the Amino acids and Sulfur classes were more abundant (23.7% and 8.1%, respectively) at E7. In comparison, those genes related to the Carbohydrates class showed the highest abundance (42.8%) at E44, the genes related to the Aromatics and Nitrogen classes were more abundant (11.3% and 3.9%, respectively) at E46, and the genes related to the Protein and Stress classes showed their highest abundance (13.7% and 9.7%, respectively) at E47. Approximately 5.5% of genes across samples resulted unclassified (Fig. 2B).

The taxonomic affiliation of unique genes was distributed among different taxa including, Acidobacteria, Actinobacteria, Bacteroidetes, Chloroflexi, Euryarchaeota, Firmicutes, Nitrospirae, Planctomycetes, Proteobacteria, Verrucomicrobia within the bacterial domain, and Thaumarchaeota within the archaeal domain. The abundance of genes affiliated to certain taxa showed differences depending on the sample (Fig. 2C). For instance, Acidobacteria and Actinobacteria genes showed their highest abundance (7.9% and 13.5%, respectively) at E7 while Bacteroidetes and Verrucomicrobia genes were more abundant genes (12.1% and 3.4%, respectively) at E46, Chloroflexi genes showed their highest abundance (8.8%) E2 and Firmicutes genes (23. 1%) at E47. In comparison, Nitrospirae and Planctomycetes genes were only observed at E46 (abundance values of 1.7% and 0.5%, respectively). Proteobacteria genes were broadly distributed among samples and accounted for up to 54.9% of the total number of unique genes at E46. And genes with no taxonomic affiliation (Fig. 2C) represented a total of 1.1%.

**Figure 3:**
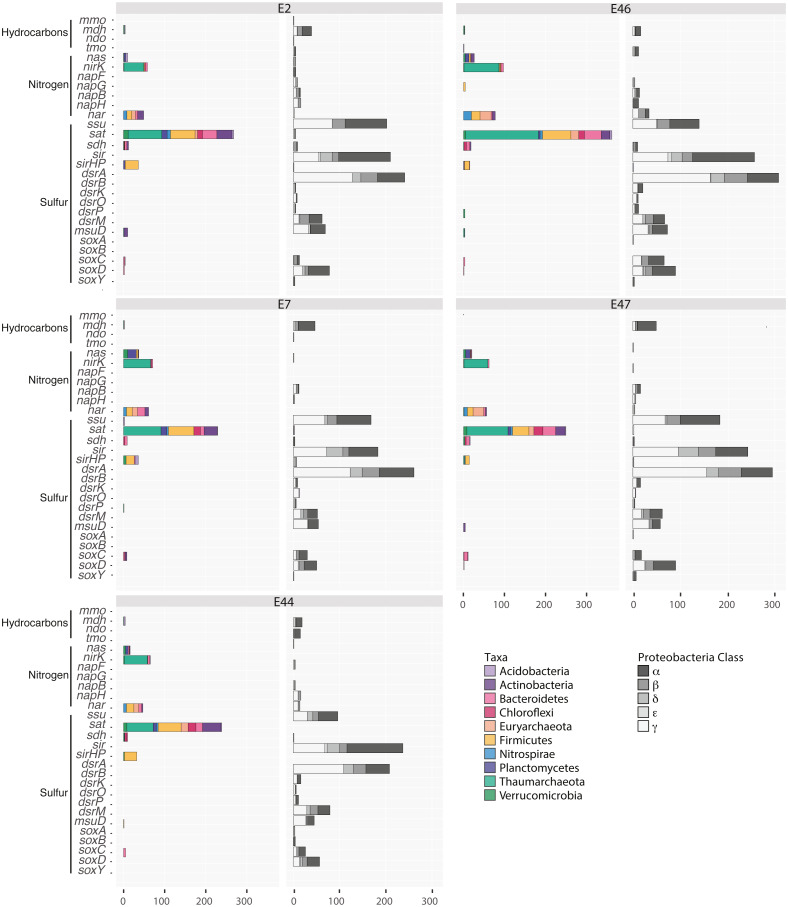
Distribution of genes related to hydrocarbon, sulfur and nitrogen metabolism in the Southern GoM sediments metagenomes (E2, E7, E44, E46 and E47). Bar plots depict the number of ORFs annotated to genes including: methane monooxygenase (MMO), methanol dehydrogenase (Mdh), naphthalene dioxygenase (NDO), toluene monooxygenase (TMO), assimilatory nitrate reductase (Nas), copper-containing nitrite reducatse (CuNiRs), ferredoxin nitrate reductase (NapBFGH), nitrate reductase cytochrome c550-type (Nar), alkane sulfonate (Ssu), sulfate adenyltransferase (Sat), sulfite oxidase (SDH), sulfite reductase (SiR), sulfite reductase [NADPH] hemoprotein Beta-component (SiRHP), sulfite sulfite reduction complex (DrsABKOPM), sulfonate monooxygenase (MsuD), sulfur oxidation protein (SoxABCDY). Bars are colored base on the taxonomic affiliation of genes according to key.

In addition, differences in the distribution of essential genes for hydrocarbon, sulfur and nitrogen metabolisms were identified. Among these, genes associated to methane oxidation to methanol (methane monooxygenase (*mmo*) and methanol dehydrogenase (*mdh*)), toluene oxidation (toluene monooxygenase (*tmo*)), and naphthalene degradation (naphthalene dioxygenase (*ndo*)); nitrite and nitrate reduction (assimilatory nitrate reductase large subunit (*nas*), copper-containing nitrite reductase (*nirK*), periplasmic ferredoxin nitrate reductase (*napBFGH*) and nitrate reductase cytochrome c550-type subunit (*nar*); sulfite reduction and oxidation (sulfite oxidase (*sdh*), sulfite reductase (*sir*), sulfite reductase [NADPH] hemoprotein Beta-component (*sirHP*), and sulfite reduction- complex(*drsABKOPM*)), sulfur oxidation (sulfur oxidation protein (*soxABCDY*)), as well as the sulfate adenylyltransferase (*sat*), the alkane sulfonate (*ssu*) and the sulfonate monooxygenase (*msuD*) were observed (Fig. 3). These genes were affiliated with diverse microbial groups including, Actinobacteria, Acidobacteria, Bacteroidetes, Chloroflexi, Firmicutes, Nitrospirae, Planctomycetes, Proteobacteria and Verrucomicrobia within the bacterial domain, and Thaumarchaeota and Euryarchaeota within the archaeal domain (Fig. 3).

Some genes peaked in abundance at specific locations and showed specific taxonomic affiliations. Namely, genes related to sulfite dehydrogenase cytochrome subunit, sulfite reductase [NADPH] hemoprotein beta-component and sulfonate monooxygenase affiliated with Bacteroidetes, Firmicutes and Actinobacteria, respectively, were more abundant at E2. In addition, assimilatory nitrate reductase genes and alkanesulfonate monooxygenase genes affiliated with Plantomycetes and Actinobacteria, respectively, were more abundant at E7 while methanol dehydrogenase genes affiliated with Acidobacteria were more abundant at E44. In comparison, genes related to toluene monooxygenase, copper-containing nitrite reductase, perisplamic ferredoxin nitrate reductase and respiratory nitrate reductase, sulfate adenyltransferase, sulfite oxidase and reductase affiliated with Actinobacteria, Bateroidetes, Planctomycetes and Verrucomicrobia, as well as Euryarchaeota and Thaumarchaeota were more abundant at E46 (Fig. 3). Moreover, some genes were exclusively affiliated to proteobacterial classes, including periplasmic ferredoxin nitrate reductase affiliated with the Rhodobacterales, Vibrionales and Campylobacterales within the Alpha, Gamma and Epsilon proteobacteria; methanol dehydrogenase affiliated with Burkholderiales, Methylophilales and Methylococcales within the Beta and Gammaproteobacterial; methane monooxygenase genes were affiliated with the Rhizobiales, Rhodobacterales and Rhodospirillales within the Alphaproteobacteria; naphthalene dioxygenase genes affiliated with the Rhizobiales within the Alphaproteobacteria; nitrate reductase cytochrome c550-type subunit affiliated with Rhizobiales, Alteromonadales, Pseudomonadales and Vibrionales within the Alpha and Gammaproteobacterial; sulfite reductase Alpha and Beta subunit, sulfite reduction complex subunits and sulfur oxidation protein subunits affiliated with Rhizobiales, Rhodospirillales, Magnetococcales, Hydrogenophilales, Rhodocyclales, Chromatiales, Oceanospirillales, Thiotrichales, Rhodobacterales, Burkholderiales, Cellvibrionales and Pseudomonadales within the Alpha, Beta and Gammaproteobacteria (Fig. 3; Table SV).

### Microbial community functional patterns

**Figure 4:**
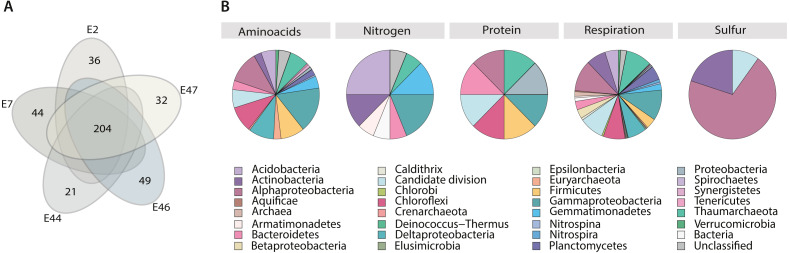
Metabolism classification and distribution in the Southern GoM sediments. (A) Five-way Venn-diagram depicts the number of shared (microbial community metabolic core) and unique pathways observed in metagenomes (E2, E7, E44, E46 and E47). (B) Pie charts show pathways classification into type of metabolism and their taxonomic distribution based on ORFs annotations within pathways. Pie charts are divided based on taxa frequency within pathway and colored as indicated in key.

In order to gain insight into the microbial community metabolic potential, predictions of metabolic pathways were performed using the pathway tools prediction algorithm in MetaPathways. A total of 655 metabolic pathways were predicted across samples showing differences on the number of reactions covered within pathways among samples (Table SVI). Further, the microbial community metabolic core was identified with a total of 204 metabolic pathways (31% of the predicted pathways) (Fig. 4A), of which 45% were fully covered (100% reactions coverage), and 23% showed more than 60% reaction coverage (Table SVI). Among the core metabolic pathways, we identified pathways related to amino acids (biosynthesis and degradation), respiration, protein, and nitrogen and sulfur metabolisms including the glycerol-3-phosphate to cytochrome bo oxidase electron transfer, glycerol-3-phosphate to fumarate electron transfer, NADH to cytochrome bd oxidase electron transfer I, NADH to cytochrome bo oxidase electron transfer I, succinate to cytochrome bd oxidase electron transfer, succinate to cytochrome bo oxidase electron transfer and aerobic respiration I (cytochrome c). The TCA cycle and the 2-oxoglutarate decarboxylation to succinyl-CoA, and pentose phosphate pathway (partial and oxidative branch) were observed, as well as the pyruvate decarboxylation to acetyl CoA I, ethanol degradation IV, formaldehyde oxidation II, and urea cycle and sulfite oxidation III pathways (Table SVI).

Core metabolic pathways were mostly distributed among all the identified taxa. However, certain pathways related to nitrogen, protein and sulfur seemed to be taxa specific, including microbial groups that were considered not abundant based on gene content alone, such as: Aquificae, Armatimonadetes, Caldithrix, Candidate divisions, Chlorobi, Deinococcus-Thermus, Elusimicrobia, Gemmatimonadetes, Spirochaetes, Synergistetes and Tenericutes, and Crenarchaeota (Fig. 4B). For example, metabolic pathways related to Nitrogen metabolism were mostly attributed to Acidobacteria, Actinobacteria, Armatimonadetes, Bacteroidetes, Deltaproteobacteria, Gemmatimonadetes and Thaumarchaeota; metabolic pathways related with protein metabolism were mostly attributed to Alphaproteobacteria, Bacteroidetes, Candidate divisions, Firmicutes, Deltaproteobacteria and Thaumarchaeota; metabolic pathways related with sulfur metabolism were mostly attributed to Alphaproteobacteria, Actinobacteria and Candidate divisions (Fig. 4B).

**Figure 5:**
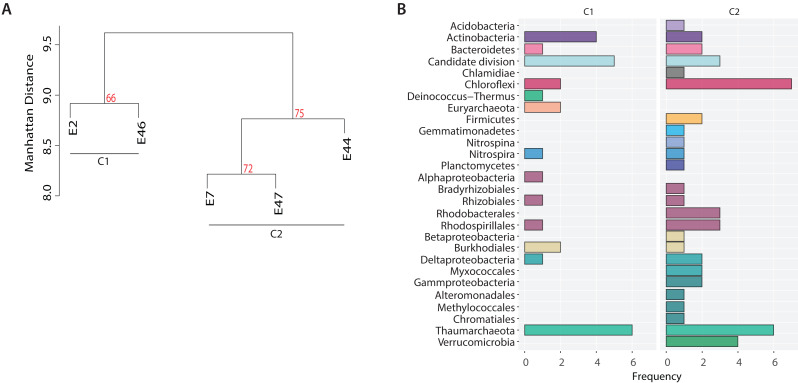
Metabolic patterns across the Southern GoM continental slope (C1) and abyssal plain (C2). (A) Hierarchical clustering of microbial community metabolic pathways based on Manhattan distance (AU > 60, 1,000 iterations). (B) Bar plots depict the number of pathways (frequency) affiliated to the microbial taxonomic groups observed in clusters.

In addition, to identify potential metabolic patterns driven by the geochemical conditions from the different locations sampled, a hierarchical clustering analysis (HCA) was conducted using the whole set of predicted pathways. Hierarchical clustering analysis indicated that the microbial community’s metabolic composition could be grouped in two major clusters (AU > 60, 1,000 iterations) associated with two GoM regions: the continental slope (cluster I: E2 and E46) and the abyssal plain (cluster II: E7, E44 and E47) (Fig. 5A). The continental slope (cluster I) was significantly correlated with terrigenous content (*R*^2^ = 0.9368, *p* < 0.1), the abyssal plain (cluster II) was significantly correlated with carbonate content (*R*^2^ = 0.9359, *p* < 0.1), while organic carbon content was not significantly correlated with any cluster (*R*^2^ = 0.1782) (Fig. 5A). Thus, distinctive metabolic pathways within samples in clusters were identified. For instance, samples from the continental slope (cluster I) were characterized by the presence of metabolic pathways such as malate/L-aspartate shuttle pathway, L-lysine biosynthesis I, formaldehyde oxidation IV (thiol-independent), L-malate degradation II, glycolysis I (from glucose 6-phosphate), Entner-Doudoroff pathway III (semi-phosphorylative), L-lysine degradation VIII, mannitol degradation I, and assimilatory sulfate reduction III. In comparison, samples from the abyssal plain (cluster II) were characterized for metabolic pathways such as methanol oxidation to formaldehyde II, nitrite oxidation, dissimilatory sulfate reduction II, glycolysis III, hydrogen oxidation II (aerobic, NAD), ammonia assimilation cycle II, methylamine degradation I, nitrate reduction IV (dissimilatory), formate oxidation to CO_2_, formate to nitrite electron transfer, manganese oxidation I, acetate and ATP formation from acetyl-CoA I, NAD salvage pathway III (to nicotinamide riboside) (Table SVII). Regarding the taxonomic distribution of these distinctive pathways, the continental slope (cluster I) showed pathways belonged to Actinobacteria, Candidate divisions, Deinococcus-Thermus, and Thaumarchaeota and Euryarchaeota , while the abyssal plain (cluster II) pathways belonged to the Acidobacteria, Firmicutes, Gemmatimonadetes, Nitrospira, Planctomycetes, Proteobacteria and Verrucomicrobia (Fig. 5B) (Table SVI).

## Discussion

This study explores metagenomic taxonomic and functional information that encompass the microbial community potential metabolism in the Southern GoM deep-sea sediments, and their likely ecological role. Our observations suggest that the prokaryotic community is constituted by a taxonomic and functional core, and that specific types of metabolisms could potentially be carried out at different locations depending on their geochemical characteristics. Our results are one of the first to provide an insight into potential metabolisms associated with specific taxa within the prokaryotic community from the Southern GoM deep-sea sediments. This represents fundamental knowledge crucial to developing future surveys based on testing and quantifying changes in nutrient cycling as a response to community’s exposure to pollutants associated with a developing oil industry in this region, and a better understanding of the ecological roles of this community.

Values for carbonates, terrigenous and organic carbon observed were consistent with the geochemical history of sediments reported for the Southern GoM region (Herzka et al., 2014). Díaz-Asencio, Bartrina & Herguera (2019) highlighted the importance of the terrigenous input to this basin, as it is one of the main controls of the burial rates with important implications on the organic matter composition and distribution in the deep-sea sediments of the Southern GoM (Díaz-Asencio, Bartrina & Herguera, 2019). In addition, for the Southern GoM, isotopic surveys have reported deep-sea sediments enriched in ^13^C content (Herzka et al., 2014); suggesting high terrigenous inputs from continental sources and a main refractory composition for the organic carbon.

Organic matter abundance and content are important variables shaping prokaryotic communities’ composition in marine sediments (Bienhold, Boetius & Ramette, 2012; Bienhold et al., 2016; D’Hondt et al., 2009; Jacob et al., 2013; Kallmeyer et al., 2012). Nonetheless the taxonomic core reported in this study is comparable to the prokaryotic groups commonly identified in deep-sea sediments using 16S rRNA gene analyses (Biddle et al., 2006; Bowman & McCuaig, 2003; Carr et al., 2015; Godoy-Lozano et al., 2018; Goffredi et al., 2008; Kimes et al., 2013; Kubo et al., 2012; Orcutt et al., 2011; Parkes et al., 2014; Polymenakou et al., 2005; Polymenakou et al., 2009; Teske, 2006) which are considered major players in nutrient cycling in marine environments (Wasmund, Muzmann & Loy, 2017), however, these taxa have also been observed in organic-poor sediments (Inagaki et al., 2003; Inagaki et al., 2006; Kormas et al., 2003; Newberry et al., 2004; Walsh et al., 2016; Webster et al., 2006). Moreover, rare (<1% relative abundance) members that may play a role in the Nitrogen, Protein and Sulfur cycling in this region were identified by gene affiliation observations. Rare taxa affiliated to some of the groups identified in this study *i.e.*, Chlorobi, Deinococcus-Thermus, Elusimicrobia, Gemmatimonadetes, Lentisphaerae, Spirochaetes and Tenericutes, have also been identified in low abundance in sediments from different aquatic environments, particularly associated with the oxic-anoxic transition in the environment (Chiellini et al., 2013; Liu et al., 2015; Martino et al., 2019; Oni et al., 2015; Rissanen et al., 2019).

To study the influence of the microbial community from the Southern GoM on possible hydrocarbon degradation processes, genes related to hydrocarbon metabolism were identified. Previous studies in marine sediments from the southwestern GoM have inferred potential hydrocarbon metabolism based on the presence of hydrocarbon degrading bacteria, mainly affiliated with genera within the Gammaproteobacteria, with well known hydrocarbon-degrading capabilities (reviewed in Rodriguez-Salazar, 2020). Hydrocarbon metabolism genes were almost exclusively found in the Alpha and Betaproteobacteria, only the toluene monooxygenase was also affiliated with Actinobacteria. Genes coding for the methane monooxygenase were affiliated with Rhizobiales, Rhodobacterales and Rhodospirillales within the Alphaproteobacteria. Our observations suggest the methanotrophic community in Southern GoM sediments, far located from oil rigs, is mostly constituted by Type II methanotrophs. Similarly, metagenomic analyses conducted following the Deepwater Horizon oil spill in the Northern GoM showed Type II methanotrophs contributed to the highest percentage (37%) of Proteobacterial genes in the sediment sample furthest located from oil rigs (∼127 Km distance) (Kimes et al., 2013). In contrast, surveys addressing hydrocarbon degradation in the Northern GoM near oil rigs (∼17 Km distance) showed the methanotrophic community was mostly constituted by Type I methanotrophs affiliated with the Gammaproteobacteria (Bacosa et al., 2018; Yergeau et al., 2015). Observations suggest methanotrophic diversity in the GoM may vary as a function of methane availability in relation to proximity to oil rigs. In addition, naphthalene dioxygenase and toluene monooxygenase were affiliated with Rhizobiales within the Alphaproteobacteria, and Burkholderiales and Rhodocyclales within the Betaproteobacteria. These orders have been previously identified as hydrocarbon and aromatic compound degraders under culture conditions (Chakraborty et al., 2005; Espinoza Tofalos et al., 2018; Martínez-Lavanchy et al., 2015; Nakatsu et al., 2006). In addition, Actinobacterial toluene monooxygenase was also identified supporting their potential ability to metabolize both *n*-alkanes and aromatic compounds, as previously reported for different actinomycete strains isolated from a wide range of sources including sediments (Chen et al., 2017; Gao et al., 2015).

Regarding nitrogen and sulfur metabolism, genes previously observed in Southwestern GoM sediments were identified (Raggi et al., 2020), including the nitrate and nitrite reductases and sulfite reductases (Fig. 3), suggesting that the microbial community in these deep-sea sediments can adapt to low oxygen conditions. Additionally, these nitrogen and sulfur genes could also be used to support alternative metabolic pathways *i.e.*, hydrocarbons and amino acids degradation. Interestingly, nitrate and nitrite reductase genes were found affiliated with the methane, naphthalene and toluene degraders identified in this study. For instance, nitrate and nitrite reductase genes were affiliated with *Bradyrhizobium* sp., within the Rhizobiales, and with *Cupriavidus metallidurans* and *Dechloromonas aromatica* within the Burkholderiales and Rhodocyclales. These observations suggest that the hydrocarbon metabolism in the microbial community from Southern GoM sediments may be coupled with nitrate and nitrite reduction as a potential alternative mechanism to cope with limiting O_2_ conditions. Namely, the use of nitrogen species to drive aerobic methane oxidation has been reported for methanotrophic communities under bioreactor, microcosm and culture conditions (Cuba et al., 2011; Hernandez et al., 2015; Kits, Klotz & Stein, 2015). Similarly, studies have demonstrated *C. metallidurans* and *D. aromatica* are capable of oxidizing toluene under nitrate reducing culture conditions (Chakraborty et al., 2005; Espinoza Tofalos et al., 2018).

Functional blueprints showed similarities among metagenomes, and these constituted a core set of metabolic pathways, which at the broadest level of gene taxonomic and functional classification were comparable to previous metagenomic observations in the Northern GoM (Kimes et al., 2013). Amino acids pathways constituted up to 20% of the core metabolism among metagenomes, and 20% of amino acids ORFs within pathways were affiliated with Sulfur reducing bacteria belonging to the Acidobacteria, Chloroflexi and Firmicutes. Further studies should be directed towards the alanine, aspartate and glutamate degradation pathways identified in this study, as they are likely to have an effect on carbon assimilation pathways, thus, amino acids may play an important role as intermediates in carbon and nitrogen cycling in these marine sediments. Some studies have characterized the role of dissolved free amino acids *i.e.*, alanine and glutamic acid in biogeochemical processes such as sulfate reduction and ammonium production by sulfate reducing bacteria in marine sediments (Burdige, 1991; Dilling & Cypionka, 2006; Jacobson, Mackin & Caponet, 1987). Culture experiments have shown sulfate reducing bacteria are capable of aerobic respiration, although not coupled with growth resulting in ATP formation (Dilling & Cypionka, 2006). In addition, results from incubation experiments amended with amino acids showed sulfate reducing bacteria can either completely oxidize them to CO_2_, or partially oxidize them to organic acids such as acetate, propionate or pyruvate (Burdige, 1991; Jacobson, Mackin & Caponet, 1987). In addition, we observed genes related to both amino acids and sulfur reducing pathways were affiliated with Acidobacteria, Chloroflexi and Firmicutes. For instance, recent surveys have shown Acidobacteria have the potential for sulfate reduction in terrestrial and aquatic ecosystems, including marine sediments (Flieder et al., 2020; Hausmann et al., 2018). Similarly, previous studies support that Chloroflexi has the metabolic capacity for amino acid catabolism and hydrocarbon degradation (Hug et al., 2013). Comparably, Firmicutes abundance has shown an increase in enrichment cultures amended with amino acids, suggesting Firmicutes has the potential for being considered amino acid utilizing bacteria (Yu et al., 2020).

Regarding central carbon assimilation pathways, we observed the TCA pathway was one of the core metabolic pathways and was highly represented across samples (80% coverage). This suggests that the TCA pathway is a preferential route for heterotrophic microorganisms to synthesize ATP and carry out a wider metabolic network contributing to other aspects of metabolism, where the reducing power generated by the TCA cycle could be utilized to regenerate NAD+ and maintain redox balance (Glöckner et al., 2003; Jenkins & Nunn, 1987; Wang et al., 2020). In addition, the aspartate, glutamate, glyoxylate and leucine degradation pathways were also part of the metabolic core across samples (>60% coverage). Results from culture-based experiments have shown these pathways could be connected to the TCA cycle as substrates to facilitate microbial growth under carbon limiting conditions such as those observed in the GoM sediments (Halsey et al., 2017; Varahan et al., 2020b; Varahan et al., 2020a; Zúñiga Ripa et al., 2014).

Moreover, statistical analyses allow us to determine region-specific clusters that may reflect differential microbial community functional patterns. In order to gain insight into potential functional patterns, clusters’ distinctive metabolic pathways were highlighted as studies have demonstrated how changes in environmental factors influence the phylogenetic diversity and functional traits of the microbial communities, and consequently ecosystem function (Baltar et al., 2016; Lucas, Wichels & Gerdts, 2016; Thompson et al., 2017; Tinta et al., 2015; Zhang, Zheng & Jiao, 2016). The Southern GoM continental slope (cluster I) is considered to be impacted by high anthropogenic pressures, specially from the oil industry and river run off, and typically display sediment contamination by polycyclic aromatic hydrocarbons and heavy metals, which in turn can result in oxidative stress with cascade effects on the marine ecosystem (Dell’Anno et al., 2021). The Entner- Doudoroff (ED) pathway was observed as one of the potential routes in the cycling of mono- and polysaccharides at the continental slope (cluster I) deep-sediments. Tracer studies have shown that the ED pathway has a preferential use in different classes of marine bacteria due to the enhanced supply of NADPH obtained and might confer other advantages for cellular metabolism *i.e.*, protection against oxidative stress (Klingner et al., 2015). Furthermore, results from recent studies suggest the ED pathway may be related to denitrification processes (Chen et al., 2020) and to novel types of sulfur metabolism (Felux et al., 2015). In addition, in the continental slope samples the assimilatory sulfate reduction pathway was also observed. This pathway is known to be coupled with the biosynthesis of sulfur-containing amino acids L-cysteine (Cys) and L-methionine (Met). Interestingly, both, L-Cys and L-Met biosynthesis were core metabolic pathways seen with at least 60% coverage in the continental slope region. The distinct redox properties of sulfur species, including Cys and Met, ensure that they play vital roles within a cell, including antioxidation and maintenance of the redox state (Tang, 2020). Therefore, the prokaryotic community of the SW GoM may carry out defensive metabolic strategies for coping with the oxidative stress occurring in the continental slope, likely due to the exposure to pollution from continental sources.

In comparison, the abyssal plain (cluster II) was characterized by metabolic pathways related to C1 metabolism, and nitrogen and sulfur cycling, suggesting the communities’ capacity for using one-carbon compounds derived from hydrocarbon sources may be a trait for growing within an otherwise carbon impoverished environment. Regarding pathways within C1 metabolism, the methanol oxidation to formaldehyde and methylamine degradation were identified. Recent observations of tracer experiments in the GoM offshore waters have shown methanol and methylamine were predominantly used as an energy source under oligotrophic conditions, in which the methylamine was suggested to be used as an alternative nitrogen source (Zhuang et al., 2018). Furthermore, contrasting evidence of nitrite oxidation and dissimilatory nitrate reduction was observed in the abyssal plain region, and supported by higher abundances of dissimilatory nitrate reductase genes compared to assimilatory nitrate reductase and copper-containing nitrite reductase (2-fold in total number of gene sequences. Although canonically, the nature of dissimilatory nitrate reduction dictates this is a process of energy conservation in the (near) absence of oxygen, studies have shown O_2_ and ammonium concentrations in sediments have an effect on the denitrification pathway carried out by the microbial community (Bonin & Raymond, 1990). For instance, results from bioreactors studies using marine sediments showed denitrification rates decreased steeply when O_2_ concentration in the pore water increased, along with an increase in ammonium possibly due to the dissimilatory nitrate reduction pathway (Bonin & Raymond, 1990). Oxygen content measurements in the Southern GoM showed oxic conditions prevailed in sediments, thus likely supporting a dissimilatory denitrification pathway to ammonium. Similarly, dissimilatory sulfate reduction was also observed in the abyssal plain region. Culture experiments have shown sulfate reduction to be possible under aerobic conditions. Results from Dilling & Cypionka (2006), showed sulfate reducing bacteria are capable of aerobic respiration, although not coupled with growth resulting in ATP formation (Dilling & Cypionka, 2006). In addition, and relevant to the community core metabolism observed in the SW GoM sediments, results from incubation experiments amended with amino acids showed sulfate reducing bacteria can either complete oxidizing them to CO_2_ or perform a partial oxidation to organic acids such as acetate, propionate or pyruvate (Burdige, 1991; Jacobson, Mackin & Caponet, 1987). Understanding these metabolic interactions may guide future development of microbial processes in new application scenarios such as bioremediation, C1 bioconversion and biocatalysis, all relevant processes to the current Gulf of Mexico ecological and economical needs.

## Conclusions

In the present study we used metagenomic analysis to determine the prokaryotic community taxonomic and functional composition relevant to understanding its potential function in the sediments of the continental slope and the abyssal plain of the Southern GoM, and in consequence, their ecological importance. Our observations highlight the metabolic potential and versatility for hydrocarbon degraders to potentially use alternative electron acceptors for hydrocarbon removal with possible effects on nitrogen cycling processes in the Southern GoM sediments, and with potential applicability to bioremediation strategies to mitigate hydrocarbon pollution in this region. Further, results suggest amino acids metabolism may play a crucial role as a central carbon source to favor growth in the southern GoM impoverished carbon environment. In addition, the prokaryotic community of the Southern GoM showed a clear division between the continental slope and abyssal plain, the former with the metabolic potential to thrive under oxidative stress conditions and the latter to cope for carbon limiting growth conditions. Our observations highlight the potential of hydrocarbon degraders, nitrate and nitrite reducers, and sulfate reducers to carry out these types of metabolism. The results presented here provide unprecedented information for potential metabolisms carried out by specific taxa in the Southern GoM sediments. This information is relevant to future studies that consider developing methodologies that can be used as microbial-based bioremediation strategies, as well as developing modeling tools that allow to predict the effects of microbial community’s response to perturbation that could feed back on ecosystem’s biogeochemical cycling, as well as for designing contingency plans and mitigation activities associated with oil-related environmental impacts.

## Supplemental Information

10.7717/peerj.12474/supp-1 Supplemental Information 1 Raw geochemical data The carbonates, particulate organic carbon and terrigenous content of each sample.

10.7717/peerj.12474/supp-2 Supplemental Information 2 Supplementary Tables

10.7717/peerj.12474/supp-3 Supplemental Information 3 Supplementary Figures

## Additional Information and Declarations

### DNA Deposition

The following information was supplied regarding the deposition of DNA sequences: Metagenome sequences data is available at EMBL-EBI: PRJEB47540.

### Data Availability

The following information was supplied regarding data availability: The data is available at MG-RAST: https://www.mg-rast.org/linkin.cgi?project=mgp13888. The other raw data are available in the Supplementary Files.

## References

[ref-1] Abella-Gutiérrez J, Herguera JC (2016). Sensitivity of carbon paleoproductivity in the Southern California Current System on different time scales for the last 2 ka. Paleoceanography.

[ref-2] Arndt S, Jørgensen BB, LaRowe DE, Middelburg JJ, Pancost RD, Regnier P (2013). Quantifying the degradation of organic matter in marine sediments: a review and synthesis. Earth-Science Reviews.

[ref-3] Bacosa HP, Erdner DL, Rosenheim BE, Shetty P, Seitz KW, Baker BJ, Liu Z (2018). Hydrocarbon degradation and response of seafloor sediment bacterial community in the northern Gulf of Mexico to light Louisiana sweet crude oil. The ISME Journal.

[ref-4] Baltar F, Currie K, Stuck E, Roosa S, Morales SE (2016). Oceanic fronts: transition zones for bacterioplankton community composition. Environmental Microbiology Reports.

[ref-5] Biddle JF, Lipp JS, Lever MA, Lloyd KG, Sørensen KB, Anderson R, Fredricks HF, Elvert M, Kelly TJ, Schrag DP, Sogin ML, Brenchley JE, Teske A, House CH, Hinrichs K-U (2006). Heterotrophic Archaea dominate sedimentary subsurface ecosystems off Peru. Proceedings of the National Academy of Sciences of the United States of America.

[ref-6] Bienhold C, Boetius A, Ramette A (2012). The energy–diversity relationship of complex bacterial communities in Arctic deep-sea sediments. The ISME Journal.

[ref-7] Bienhold C, Zinger L, Boetius A, Ramette A (2016). Diversity and biogeography of bathyal and abyssal seafloor bacteria. PLOS ONE.

[ref-8] Bik HM, Halanych KM, Sharma J, Thomas WK (2012). Dramatic shifts in benthic microbial eukaryote communities following the Deepwater horizon oil spill. PLOS ONE.

[ref-9] Bonin P, Raymond N (1990). Effects of oxygen on denitrification in marine sediments. Hydrobiologia.

[ref-10] Bowman JP, McCuaig RD (2003). Biodiversity, community structural shifts, and biogeography of prokaryotes within Antarctic continental shelf sediment. Applied and Environmental Microbiology.

[ref-11] Burdige DJ (1991). Microbial processes affecting alanine and glutamic acid in anoxic marine sediments. FEMS Microbiology Letters.

[ref-12] Burdige DJ (2007). Preservation of organic matter in marine sediments: controls, mechanisms, and an imbalance in sediment organic carbon budgets?. Chemical Reviews.

[ref-13] Carr SA, Orcutt BN, Mandernack KW, Spear JR (2015). Abundant Atribacteria in deep marine sediment from the Adélie Basin, Antarctica. Frontiers in Microbiology.

[ref-14] Caspi R, Billington R, Ferrer L, Foerster H, Fulcher CA, Keseler IM, Kothari A, Krummenacker M, Latendresse M, Mueller LA, Ong Q, Paley S, Subhraveti P, Weaver DS, Karp PD (2016). The MetaCyc database of metabolic pathways and enzymes and the BioCyc collection of pathway/genome databases. Nucleic Acids Research.

[ref-15] Chakraborty R, O’Connor SM, Chan E, Coates JD (2005). Anaerobic degradation of benzene, toluene, ethylbenzene, and xylene compounds by *Dechloromonas* strain RCB. Applied and environmental microbiology.

[ref-16] Chen H, Boutros PC (2011). VennDiagram: a package for the generation of highly-customizable Venn and Euler diagrams in R. BMC Bioinformatics.

[ref-17] Chen S, Li S, Huang T, Yang S, Liu K, Ma B, Shi Y, Miao Y (2020). Nitrate reduction by *Paracoccus thiophilus* strain LSL 251 under aerobic condition: performance and intracellular central carbon flux pathways. Bioresource Technology.

[ref-18] Chen W, Li J, Sun X, Min J, Hu X (2017). High efficiency degradation of alkanes and crude oil by a salt-tolerant bacterium *Dietzia* species CN-3. International Biodeterioration & Biodegradation.

[ref-19] Chiellini C, Iannelli R, Verni F, Petroni G (2013). Bacterial communities in polluted seabed sediments: a molecular biology assay in Leghorn Harbor. The Scientific World Journal.

[ref-20] Cuba RMF, Duarte IC, Saavedra NK, Varesche MBA, Foresti E (2011). Denitrification coupled with methane anoxic oxidation and microbial community involved identification. Brazilian Archives of Biology and Technology.

[ref-21] Dell’Anno F, Rastelli E, Tangherlini M, Corinaldesi C, Sansone C, Brunet C, Balzano S, Ianora A, Musco L, Montereali MR, Dell’Anno A (2021). Highly contaminated marine sediments can host rare bacterial taxa potentially useful for bioremediation. Frontiers in Microbiology.

[ref-22] D’Hondt S, Inagaki F, Zarikian CA, Abrams LJ, Dubois N, Engelhardt T, Ziebis W (2015). Presence of oxygen and aerobic communities from sea floor to basement in deep-sea sediments. Nature Geoscience.

[ref-23] D’Hondt S, Spivack AJ, Pockalny R, Ferdelman TG, Fischer JP, Kallmeyer J, Abrams LJ, Smith DC, Graham D, Hasiuk F, Schrum H, Stancin AM (2009). Subseafloor sedimentary life in the South Pacific Gyre. Proceedings of the National Academy of Sciences of the United States of America.

[ref-24] Díaz-Asencio M, Bartrina VF, Herguera JC (2019). Sediment accumulation patterns on the slopes and abyssal plain of the southern Gulf of Mexico. Deep Sea Research Part I: Oceanographic Research Papers.

[ref-25] Dilling W, Cypionka H (2006). Aerobic respiration in sulfate-reducing bacteria. FEMS Microbiology Letters.

[ref-26] Espinoza Tofalos A, Daghio M, González M, Papacchini M, Franzetti A, Seeger M (2018). Toluene degradation by cupriavidus metallidurans CH34 in nitrate-reducing conditions and in bioelectrochemical systems. FEMS Microbiology Letters.

[ref-27] Fautin D, Dalton P, Incze LS, Leong J-AC, Pautzke C, Rosenberg A, Sandifer P, Sedberry G, Tunnell Jr JW, Abbott I, Brainard RE, Brodeur M, Eldredge LG, Feldman M, Moretzsohn F, Vroom PS, Wainstein M, Wolff N (2010). An overview of marine biodiversity in United States waters. PLOS ONE.

[ref-28] Felux A-K, Spiteller D, Klebensberger J, Schleheck D (2015). Entner–Doudoroff pathway for sulfoquinovose degradation in *Pseudomonas putida* SQ1. Proceedings of the National Academy of Sciences of the United States of America.

[ref-29] Flieder M, Buongiorno J, Herbold CW, Hausmann B, Rattei T, Lloyd KG, Loy A, Wasmund K (2020). Novel taxa of Acidobacteriota involved in seafloor sulfur cycling.

[ref-30] Gao X, Gao W, Cui Z, Han B, Yang P, Sun C, Zheng L (2015). Biodiversity and degradation potential of oil-degrading bacteria isolated from deep-sea sediments of South Mid-Atlantic Ridge. Marine Pollution Bulletin.

[ref-31] Glöckner FO, Kube M, Bauer M, Teeling H, Lombardot T, Ludwig W, Gade D, Beck A, Borzym K, Heitmann K, Rabus R, Schlesner H, Amann R, Reinhardt R (2003). Complete genome sequence of the marine planctomycete *Pirellula* sp. strain 1. Proceedings of the National Academy of Sciences of the United States of America.

[ref-32] Godoy-Lozano EE, Escobar-Zepeda A, Raggi L, Merino E, Gutierrez-Rios RM, Juarez K, Segovia L, Licea-Navarro AF, Gracia A, Sanchez-Flores A, Pardo-Lopez L (2018). Bacterial diversity and the geochemical landscape in the Southwestern Gulf of Mexico. Frontiers in Microbiology.

[ref-33] Goffredi SK, Wilpiszeski R, Lee R, Orphan VJ (2008). Temporal evolution of methane cycling and phylogenetic diversity of archaea in sediments from a deep-sea whale-fall in Monterey Canyon, California. The ISME Journal.

[ref-34] Halsey CR, Lei S, Wax JK, Lehman MK, Nuxoll AS, Steinke L, Sadykov M, Powers R, Fey PD (2017). Amino Acid Catabolism in *Staphylococcus aureus* and the function of carbon catabolite repression. mBio.

[ref-35] Hausmann B, Pelikan C, Herbold CW, Köstlbacher S, Albertsen M, Eichorst SA, Glavinadel Rio T, Huemer M, Nielsen PH, Rattei T, Stingl U, Tringe SG, Trojan D, Wentrup C, Woebken D, Pester M, Loy A (2018). Peatland Acidobacteria with a dissimilatory sulfur metabolism. The ISME Journal.

[ref-36] Hedges JI, Oades JM (1997). Comparative organic geochemistries of soils and marine sediments. Organic Geochemistry.

[ref-37] Herguera García JC, Díaz Asencio M, Cepeda Morales J, Herzka RA, Zaragoza Álvarez E, Peters M, Hernández Cárdenas G (2021). Hidrografía, biogeoquímica e imágenes de satélite. Atlas de línea base ambiental del Golfo de México.

[ref-38] Hernandez ME, Beck DAC, Lidstrom ME, Chistoserdova L (2015). Oxygen availability is a major factor in determining the composition of microbial communities involved in methane oxidation. PeerJ.

[ref-39] Herzka S, Herguera JC, Licea A, Sheinbaum J, Ferreira V, Camacho V, Díaz V, Farber J, García J, Hernández Ayón M, Huerta MA, Lara R, Lares L, Lizárraga L, Macias V, Millán E, Riquelme M, Rocha A (2014). Fase III para el monitoreo ambiental en aguas profundas del Golfo de México en respuesta al derrame petrolero asociado a la plataforma Deepwater horizon.

[ref-40] Hug LA, Castelle CJ, Wrighton KC, Thomas BC, Sharon I, Frischkorn KR, Williams KH, Tringe SG, Banfield JF (2013). Community genomic analyses constrain the distribution of metabolic traits across the Chloroflexi phylum and indicate roles in sediment carbon cycling. Microbiome.

[ref-41] Inagaki F, Nunoura T, Nakagawa S, Teske A, Lever M, Lauer A, Suzuki M, Takai K, Delwiche M, Colwell FS, Nealson KH, Horikoshi K, D’Hondt S, Jørgensen BB (2006). Biogeographical distribution and diversity of microbes in methane hydrate-bearing deep marine sediments on the Pacific Ocean Margin. Proceedings of the National Academy of Sciences of the United States of America.

[ref-42] Inagaki F, Takai K, Kobayashi H, Nealson KH, Horikoshi K (2003). Sulfurimonas autotrophica gen. nov. sp. nov. a novel sulfur-oxidizing -proteobacterium isolated from hydrothermal sediments in the Mid-Okinawa Trough. International Journal of Systematic and Evolutionary Microbiology.

[ref-43] Jacob M, Soltwedel T, Boetius A, Ramette A (2013). Biogeography of Deep-Sea Benthic Bacteria at Regional Scale (LTER HAUSGARTEN, Fram Strait, Arctic). PLOS ONE.

[ref-44] Jacobson ME, Mackin JE, Caponet DG (1987). Ammonium production in sediments inhibited with molybdate: implications for the sources of ammonium in anoxic marine sediments. Applied and Environmental Microbiology.

[ref-45] Jenkins LS, Nunn WD (1987). Genetic and molecular characterization of the genes involved in short-chain fatty acid degradation in Escherichia coli: the ato system. Journal of Bacteriology.

[ref-46] Kallmeyer J, Pockalny R, Adhikari RR, Smith DC, D’Hondt S (2012). Global distribution of microbial abundance and biomass in subseafloor sediment. Proceedings of the National Academy of Sciences of the United States of America.

[ref-47] Karp PD, Paley SM, Krummenacker M, Latendresse M, Dale JM, Lee TJ, Kaipa P, Gilham F, Spaulding A, Popescu L, Altman T, Paulsen I, Keseler IM, Caspi R (2010). Pathway tools version 13.0: integrated software for pathway/genome informatics and systems biology. Briefings in Bioinformatics.

[ref-48] Karp PD, Paley S, Romero P (2002). The pathway tools software. Bioinformatics.

[ref-49] Karp PD, Riley M, Saier M, Paulsen IT, Paley SM, Pellegrini-Toole A (2000). The EcoCyc and MetaCyc databases. Nucleic Acids Research.

[ref-50] Keegan KP, Glass EM, Meyer F, Martin F, Uroz S (2016). MG-RAST, a metagenomics service for analysis of microbial community structure and function. Microbial environmental genomics (MEG).

[ref-51] Kent WJ (2002). BLAT–the BLAST-like alignment tool. Genome Research.

[ref-52] Kimes N, Callaghan A, Aktas D, Smith W, Sunner J, Golding B, Drozdowska M, Hazen T, Suflita J, Morris P (2013). Metagenomic analysis and metabolite profiling of deep–sea sediments from the Gulf of Mexico following the Deepwater horizon oil spill. Frontiers in Microbiology.

[ref-53] Kits KD, Klotz MG, Stein LY (2015). Methane oxidation coupled to nitrate reduction under hypoxia by the Gammaproteobacterium Methylomonas denitrificans, sp. nov. type strain FJG1. Environmental Microbiology.

[ref-54] Klingner A, Bartsch A, Dogs M, Wagner-Döbler I, Jahn D, Simon M, Brinkhoff T, Becker J, Wittmann C (2015). Large-Scale 13C flux profiling reveals conservation of the Entner-Doudoroff pathway as a glycolytic strategy among marine bacteria that use glucose. Applied and Environmental Microbiology.

[ref-55] Konwar KM, Hanson NW, Pagé AP, Hallam SJ (2013). MetaPathways: a modular pipeline for constructing pathway/genome databases from environmental sequence information. BMC Bioinformatics.

[ref-56] Kormas KAR, Smith DC, Edgcomb V, Teske A (2003). Molecular analysis of deep subsurface microbial communities in Nankai Trough sediments (ODP Leg 190, Site 1176). FEMS Microbiology Ecology.

[ref-57] Kubo K, Lloyd KG, Biddle JF, Amann R, Teske A, Knittel K (2012). Archaea of the miscellaneous Crenarchaeotal Group are abundant, diverse and widespread in marine sediments. The ISME Journal.

[ref-58] Liu X, Hu H-W, Liu Y-R, Xiao K-Q, Cheng F-S, Li J, Xiao T (2015). Bacterial composition and spatiotemporal variation in sediments of Jiaozhou Bay. China. Journal of Soils and Sediments.

[ref-59] Lucas J, Wichels A, Gerdts G (2016). Spatiotemporal variation of the bacterioplankton community in the German Bight: from estuarine to offshore regions. Helgoland Marine Research.

[ref-60] Martínez-Lavanchy PM, Chen Z, Lünsmann V, Marin-Cevada V, Vilchez-Vargas R, Pieper DH, Reiche N, Kappelmeyer U, Imparato V, Junca H, Nijenhuis I, Müller JA, Kuschk P, Heipieper HJ (2015). Microbial toluene removal in hypoxic model constructed wetlands occurs predominantly via the Ring Monooxygenation Pathway. Applied and Environmental Microbiology.

[ref-61] Martino A, Rhodes ME, León-Zayas R, Valente IE, Biddle JF, House CH (2019). Microbial diversity in sub-seafloor sediments from the Costa Rica margin. Geosciences.

[ref-62] Mason OU, Scott NM, Gonzalez A, Robbins-Pianka A, Bælum J, Kimbrel J, Bouskill NJ, Prestat E, Borglin S, Joyner DC, Fortney JL, Jurelevicius D, Stringfellow WT, Alvarez-Cohen L, Hazen TC, Knight R, Gilbert JA, Jansson JK (2014). Metagenomics reveals sediment microbial community response to Deepwater Horizon oil spill. The ISME Journal.

[ref-63] Middelburg JJ (2018). Reviews and syntheses: to the bottom of carbon processing at the seafloor. Biogeosciences.

[ref-64] Mitchell KR, Takacs-Vesbach CD (2008). A comparison of methods for total community DNA preservation and extraction from various thermal environments. Journal of Industrial Microbiology & Biotechnology.

[ref-65] Nakatsu CH, Hristova K, Hanada S, Meng X-Y, Hanson JR, Scow KM, Kamagata Y (2006). Methylibium petroleiphilum gen. nov. sp. nov. a novel methyl tert-butyl ether-degrading methylotroph of the Betaproteobacteria. International Journal of Systematic and Evolutionary Microbiology.

[ref-66] Newberry CJ, Webster G, Cragg BA, Parkes RJ, Weightman AJ, Fry JC (2004). Diversity of prokaryotes and methanogenesis in deep subsurface sediments from the Nankai Trough, Ocean Drilling Program Leg 190. Environmental Microbiology.

[ref-67] O’Leary NA, Wright MW, Brister JR, Ciufo S, Haddad D, McVeigh R, Rajput B, Robbertse B, Smith-White B, Ako-Adjei D, Astashyn A, Badretdin A, Bao Y, Blinkova O, Brover V, Chetvernin V, Choi J, Cox E, Ermolaeva O, Farrell CM, Goldfarb T, Gupta T, Haft D, Hatcher E, Hlavina W, Joardar VS, Kodali VK, Li W, Maglott D, Masterson P, McGarvey KM, Murphy MR, O’Neill K, Pujar S, Rangwala SH, Rausch D, Riddick LD, Schoch C, Shkeda A, Storz SS, Sun H, Thibaud-Nissen F, Tolstoy I, Tully RE, Vatsan AR, Wallin C, Webb D, Wu W, Landrum MJ, Kimchi A, Tatusova T, DiCuccio M, Kitts P, Murphy TD, Pruitt KD (2016). Reference sequence (RefSeq) database at NCBI: current status, taxonomic expansion, and functional annotation. Nucleic Acids Research.

[ref-68] Oni OE, Schmidt F, Miyatake T, Kasten S, Witt M, Hinrichs K-U, Friedrich MW (2015). Microbial communities and organic matter composition in surface and subsurface sediments of the Helgoland Mud Area, North Sea. Frontiers in Microbiology.

[ref-69] Orcutt BN, Sylvan JB, Knab NJ, Edwards KJ (2011). Microbial ecology of the dark ocean above, at, and below the seafloor. Microbiology and Molecular Biology Reviews.

[ref-70] Orsi WD (2018). Ecology and evolution of seafloor and subseafloor microbial communities. Nature Reviews Microbiology.

[ref-71] Parkes RJ, Cragg B, Roussel E, Webster G, Weightman A, Sass H (2014). A review of prokaryotic populations and processes in sub-seafloor sediments, including biosphere:geosphere interactions. Marine Geology.

[ref-72] Peterson CH, Coleman FC, Jackson JBC, Turner RE, Rowe GT, Barber RT, Bjorndal KA, Carney RS, Cowen RK, Hoekstra JM, Hollibaugh JT, Laska SB, Luettich RA, Osenberg CW, Roady SE, Senner S, Teal JM, Wang P (2011). A once and future gulf of mexico ecosystem. Restoration recommendations of an expert working group.

[ref-73] Polymenakou PN, Bertilsson S, Tselepides A, Stephanou EG (2005). Bacterial community composition in different sediments from the Eastern Mediterranean Sea: a comparison of four 16S ribosomal DNA clone libraries. Microbial Ecology.

[ref-74] Polymenakou PN, Lampadariou N, Mandalakis M, Tselepides A (2009). Phylogenetic diversity of sediment bacteria from the southern Cretan margin, Eastern Mediterranean Sea. Systematic and Applied Microbiology.

[ref-75] Raggi L, García-Guevara F, Godoy-Lozano EE, Martínez-Santana A, Escobar-Zepeda A, Gutierrez-Rios RM, Loza A, Merino E, Sanchez-Flores A, Licea-Navarro A, Pardo-Lopez L, Segovia L, Juarez K (2020). Metagenomic profiling and microbial metabolic potential of Perdido Fold Belt (NW) and Campeche Knolls (SE) in the Gulf of Mexico. Frontiers in Microbiology.

[ref-76] R Core Team (2013). http://www.R-project.org/.

[ref-77] Rissanen AJ, Peura S, Mpamah PA, Taipale S, Tiirola M, Biasi C, Mäki A, Nykänen H (2019). Vertical stratification of bacteria and archaea in sediments of a small boreal humic lake. FEMS Microbiology Letters.

[ref-78] Rodríguez-Salazar J, Loza A, Ornelas-Ocampo K, Gutierrez-Rios RM, Pardo-López L (2021). Bacteria from the Southern Gulf of Mexico: baseline, diversity, hydrocarbon-degrading potential and future applications. Frontiers in Marine Science.

[ref-79] Schnitzer M, Sparks DL (1999). A lifetime perspective on the chemistry of soil organic matter. Advances in agronomy.

[ref-80] Suzuki R, Terada Y, Shimodaira H (2015). pvclust: Hierarchical clustering with *P*-Values via multiscale bootstrap resampling. https://cran.r-project.org/web/packages/pvclust/index.html.

[ref-81] Tang K (2020). Chemical diversity and biochemical transformation of biogenic organic sulfur in the ocean. Frontiers in Marine Science.

[ref-82] Teske AP (2006). Microbial communities of deep marine subsurface sediments: molecular and cultivation surveys. Geomicrobiology Journal.

[ref-83] Thompson LR, Williams GJ, Haroon MF, Shibl A, Larsen P, Shorenstein J, Knight R, Stingl U (2017). Metagenomic covariation along densely sampled environmental gradients in the Red Sea. The ISME Journal.

[ref-84] Tinta T, Vojvoda J, Mozetič P, Talaber I, Vodopivec M, Malfatti F, Turk V (2015). Bacterial community shift is induced by dynamic environmental parameters in a changing coastal ecosystem (northern Adriatic, northeastern Mediterranean Sea) –a 2-year time-series study. Environmental Microbiology.

[ref-85] Tunnell Jr J (2009). The Gulf of Mexico. Ocean: an illustrated atlas.

[ref-86] Urakawa H, Garcia JC, Barreto PD, Molina GA, Barreto JC (2012). A sensitive crude oil bioassay indicates that oil spills potentially induce a change of major nitrifying prokaryotes from the Archaea to the Bacteria. Environmental Pollution.

[ref-87] Varahan S, Sinha V, Walvekar A, Krishna S, Laxman S (2020a). Resource plasticity-driven carbon-nitrogen budgeting enables specialization and division of labor in a clonal community. eLife.

[ref-88] Varahan S, Sinha V, Walvekar A, Krishna S, Laxman S (2020b). Metabolite plasticity drives carbon-nitrogen resource budgeting to enable division of labor in a clonal community.

[ref-89] Vargas-Gastélum L, Chong-Robles J, Lago-Lestón A, Darcy JL, Amend AS, Riquelme M (2019). Targeted ITS1 sequencing unravels the mycodiversity of deep-sea sediments from the Gulf of Mexico. Environmental Microbiology.

[ref-90] Walsh EA, Kirkpatrick JB, Rutherford SD, Smith DC, Sogin M, D’Hondt S (2016). Bacterial diversity and community composition from seasurface to subseafloor. The ISME Journal.

[ref-91] Wang S, Yan Z, Wang P, Zheng X, Fan J (2020). Comparative metagenomics reveals the microbial diversity and metabolic potentials in the sediments and surrounding seawaters of Qinhuangdao mariculture area. PLOS ONE.

[ref-92] Ward CH, Tunnell JW, Ward CH (2017). Habitats and biota of the Gulf of Mexico: an overview. Habitats and Biota of the Gulf of Mexico: before the deepwater horizon oil spill: volume 1: water quality, sediments, sediment contaminants, oil and gas seeps, coastal habitats, offshore plankton and benthos, and shellfish.

[ref-93] Wasmund K, Muzmann M, Loy A (2017). The life sulfuric: microbial ecology of sulfur cycling in marine sediments. Environmental Microbiology Reports.

[ref-94] Webster G, John Parkes R, Cragg BA, Newberry CJ, Weightman AJ, Fry JC (2006). Prokaryotic community composition and biogeochemical processes in deep subseafloor sediments from the Peru Margin. FEMS Microbiology Ecology.

[ref-95] Wickham H (2009). ggplot2: Elegant graphics for data analysis.

[ref-96] Wickham H, Averick M, Bryan J, Chang W, McGowan LD’A, François R, Grolemund G, Hayes A, Henry L, Hester J, Kuhn M, Pedersen TL, Miller E, Bache SM, Müller K, Ooms J, Robinson D, Seidel DP, Spinu V, Takahashi K, Vaughan D, Wilke C, Woo K, Yutani H (2019). Welcome to the tidyverse. Journal of Open Source Software.

[ref-97] Wickham H, François R, Henry L, Müller K (2018). dplyr: a grammar of data manipulation.

[ref-98] Wilke A, Harrison T, Wilkening J, Field D, Glass EM, Kyrpides N, Mavrommatis K, Meyer F (2012). The M5nr: a novel non-redundant database containing protein sequences and annotations from multiple sources and associated tools. BMC Bioinformatics.

[ref-99] Yergeau E, Lawrence JR, Waiser MJ, Korber DR, Greer CW (2010). Metatranscriptomic analysis of the response of river biofilms to pharmaceutical products, using anonymous DNA microarrays. Applied and Environmental Microbiology.

[ref-100] Yergeau E, Maynard C, Sanschagrin S, Champagne J, Juck D, Lee K, Greer CW (2015). Microbial community composition, functions, and activities in the Gulf of Mexico 1 Year after the Deepwater horizon accident. Applied and Environmental Microbiology.

[ref-101] Yergeau E, Sanschagrin S, Waiser MJ, Lawrence JR, Greer CW (2012). Sub-inhibitory concentrations of different pharmaceutical products affect the meta-transcriptome of river biofilm communities cultivated in rotating annular reactors. Environmental Microbiology Reports.

[ref-102] Yoskowitz D, Leon C, Gibeaut J, Lupher B, Lopez M, Santos C, Sutton G, McKinney L (2013). Gulf 360: state of the Gulf of Mexico. Harte Research Institute for Gulf of Mexico Studies.

[ref-103] Yu Y, Yang J, Zheng L-Y, Sheng Q, Li C-Y, Wang M, Zhang X-Y, McMinn A, Zhang Y-Z, Song X-Y, Chen X-L (2020). Diversity of D-amino acid utilizing bacteria from Kongsfjorden, Arctic and the Metabolic Pathways for Seven D-Amino Acids. Frontiers in Microbiology.

[ref-104] Zhang Z, Zheng Q, Jiao N (2016). Microbial D-amino acids and marine carbon storage. Science China Earth Sciences.

[ref-105] Zhuang G-C, Peña Montenegro TD, Montgomery A, Hunter KS, Joye SB (2018). Microbial metabolism of methanol and methylamine in the Gulf of Mexico: insight into marine carbon and nitrogen cycling. Environmental Microbiology.

[ref-106] Zúñiga Ripa A, Barbier T, Conde-Álvarez R, Martínez-Gómez E, Palacios-Chaves L, Gil-Ramírez Y, Grilló MJ, Letesson J-J, Iriarte M, Moriyón I (2014). Brucella abortus depends on Pyruvate Phosphate Dikinase and Malic Enzyme but not on Fbp and GlpX Fructose-1, 6-Bisphosphatases for full virulence in laboratory models. Journal of Bacteriology.

