# Supplementary material for: The metabolic core of the prokaryotic community from deep-sea sediments of the southern Gulf of Mexico shows different functional signatures between the continental slope and abyssal plain
Source: PeerJ. 2021 Dec 14;9:e12474. doi: 10.7717/peerj.12474 (PMC8679910; doi:10.7717/peerj.12474)
Supplement: Supplemental Information 3 [file peerj-09-12474-s003.docx]

**Supplementary Information**

**The metabolic core of the prokaryotic community from** **deep-sea sediments of the southern Gulf of Mexico shows different functional signatures between the continental slope and abyssal plain.**

Mónica Torres-Beltrán^1^, Lluvia B. Vargas-Gastelum^2^, Dante A. Magdaleno-Moncayo^3^, Meritxell Riquelme^2^, Juan Carlos Herguera-Garcia^4^, Alejandra Prieto-Davó^5^ and Asunción Lago-Lestón^1*^

* Corresponding author

1. **Supplementary Figures**


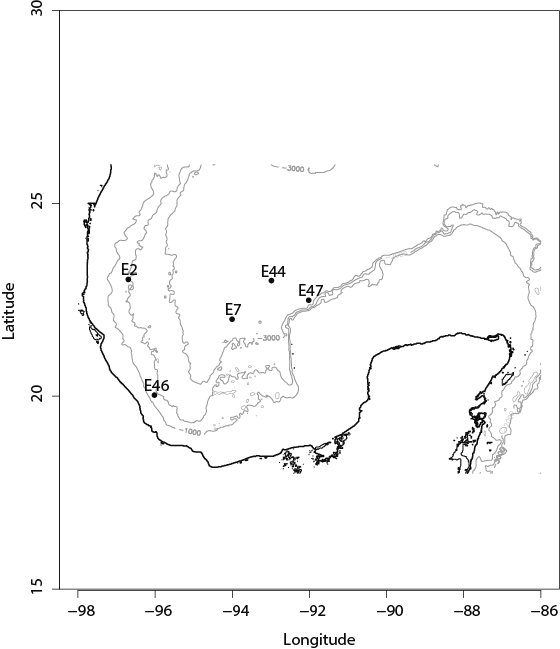


**Figure 1.** Southern Gulf of Mexico (GoM) region (25 ̊N to 19 ̊N and 87 ̊W to 95 ̊W). Samples (E2, E7, E44, E46 and E47) location is depicted as dots on map. Dark line shows the continental margin, while gray lines show the 1000, 2000 and 3000m isobaths.


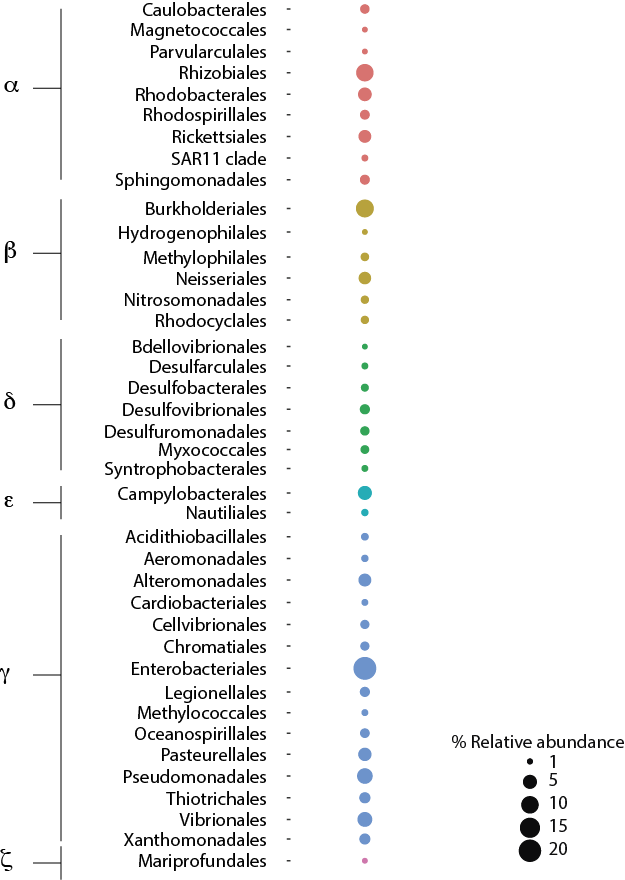


**Figure 2.** Phylum Proteobacteria breakdown into Classes and Orders. Size of dots depict the relative abundance (%) of the total number of genes affiliated to a specific Order calculated from the total number of genes in metagenomes.


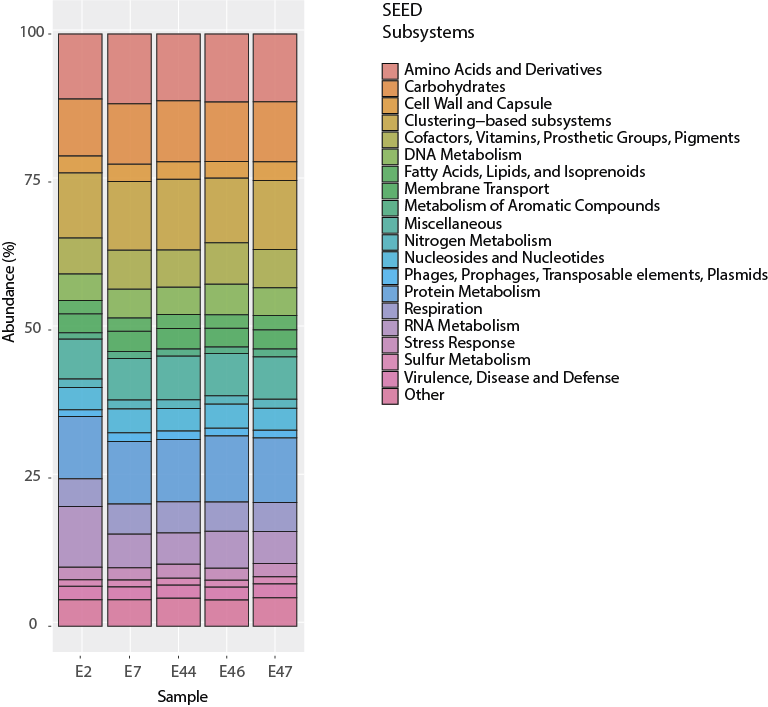


**Figure 3.** SEED Subsystems classification into categories of metabolism for Southern GoM metagenomes. Bar plots depict the relative abundance (%) of SEED Subsystems in samples (E2, E7, E44, E46 and E47). SEED Subsystems categories are color coded as indicated in color key.
